# Structure and architecture of immature and mature murine leukemia virus capsids

**DOI:** 10.1073/pnas.1811580115

**Published:** 2018-11-26

**Authors:** Kun Qu, Bärbel Glass, Michal Doležal, Florian K. M. Schur, Brice Murciano, Alan Rein, Michaela Rumlová, Tomáš Ruml, Hans-Georg Kräusslich, John A. G. Briggs

**Affiliations:** ^a^Structural and Computational Biology Unit, European Molecular Biology Laboratory, 69117 Heidelberg, Germany;; ^b^Molecular Medicine Partnership Unit, European Molecular Biology Laboratory and Universitätsklinikum Heidelberg, 69117 Heidelberg, Germany;; ^c^Structural Studies Division, Medical Research Council Laboratory of Molecular Biology, CB2 0QH Cambridge, United Kingdom;; ^d^Department of Infectious Diseases, Virology, Universitätsklinikum Heidelberg, 69120 Heidelberg, Germany;; ^e^Institute of Organic Chemistry and Biochemistry of the Czech Academy of Sciences, 16610 Prague 6, Czech Republic;; ^f^Institute of Science and Technology Austria, A-3400 Klosterneuburg, Austria;; ^g^HIV Dynamics and Replication Program, Center for Cancer Research, National Cancer Institute, Frederick, MD 21702;; ^h^Department of Biotechnology, University of Chemistry and Technology, 16628 Prague 6, Czech Republic;; ^i^Department of Biochemistry and Microbiology, University of Chemistry and Technology, 16628 Prague 6, Czech Republic

**Keywords:** murine leukemia virus, retrovirus, cryoelectron tomography, capsid, maturation

## Abstract

Retroviruses assemble and bud from infected cells in an immature form and require proteolytic maturation for infectivity. The CA (capsid) domains of the Gag polyproteins assemble a protein lattice as a truncated sphere in the immature virion. Proteolytic cleavage of Gag induces dramatic structural rearrangements; a subset of cleaved CA subsequently assembles into the mature core, whose architecture varies among retroviruses. Murine leukemia virus (MLV) is the prototypical γ-retrovirus and serves as the basis of retroviral vectors, but the structure of the MLV CA layer is unknown. Here we have combined X-ray crystallography with cryoelectron tomography to determine the structures of immature and mature MLV CA layers within authentic viral particles. This reveals the structural changes associated with maturation, and, by comparison with HIV-1, uncovers conserved and variable features. In contrast to HIV-1, most MLV CA is used for assembly of the mature core, which adopts variable, multilayered morphologies and does not form a closed structure. Unlike in HIV-1, there is similarity between protein–protein interfaces in the immature MLV CA layer and those in the mature CA layer, and structural maturation of MLV could be achieved through domain rotations that largely maintain hexameric interactions. Nevertheless, the dramatic architectural change on maturation indicates that extensive disassembly and reassembly are required for mature core growth. The core morphology suggests that wrapping of the genome in CA sheets may be sufficient to protect the MLV ribonucleoprotein during cell entry.

## Significance

Immature retroviruses are built by the Gag polyprotein; Gag is then cut into domains, and the resulting CA capsid proteins form the mature capsid, which can mediate infection of a new cell. Murine leukemia virus (MLV) is a model retrovirus and the basis for gene-delivery vectors. We determined the capsid structures and architectures for immature and mature MLV. The mature MLV core does not enclose the genome in a closed capsid by using only part of the available proteins, as is the case for HIV-1. Instead, it wraps the genome in curved sheets incorporating most CA proteins. Retroviruses therefore have fundamentally different modes of core assembly and genome protection, which may relate to differences in their early replication.

Retroviruses comprise a diverse family of enveloped RNA viruses with the hallmark of reverse transcription of their RNA genome during virus replication. *Retroviridae* are classified into the subfamilies of ortho- and spumaretroviruses, with orthoretroviruses comprising the genera α-, β-, γ-, δ-, and ε-retroviruses as well as the lentiviruses (1). For virus production, orthoretroviruses initially form an immature spherical protein shell containing the genome and replication proteins at the plasma membrane or in the cytoplasm of the infected cell. After or during envelopment and release of the complete virus from the cell, this immature structure undergoes proteolytic maturation to form the core of the mature, infectious virion (2, 3). Intermediate- or high-resolution structures have been determined for the immature capsids within a β-retrovirus [Mason–Pfizer monkey virus (M-PMV) (4)] and lentivirus [HIV-1 (4, 5)] as well as for virus-like particles mimicking an immature α-retrovirus [Rous sarcoma virus (RSV) (6)]. A structure at subnanometer resolution has also been obtained for the mature capsid within HIV-1 particles (7), which is very similar to previously described in vitro assembled arrays of CA protein (8, 9). Murine leukemia virus (MLV), the prototypic member of the γ-retroviruses, is commonly used in comparative studies and as a basis for retroviral vector design. Immature-like MLV particles assembled in vitro from purified protein have been studied at low resolution by cryoelectron tomography (cryo-ET) (10), and immature and mature MLV particles were imaged 20 y ago in two dimensions by cryo-EM (11). However, there is currently insufficient information to allow the 3D capsid architecture or the CA lattice structure of γ-retroviruses to be described.

Retrovirus assembly is driven by oligomerization of the Gag polyprotein, which forms the immature protein shell. Gag polyproteins from all retroviral genera contain the following three structural domains with conserved function: the N-terminal MA (matrix) domain, the central CA domain, and the downstream NC (nucleocapsid) domain. These domains form concentric layers in the immature Gag sphere. The domains have low sequence homology among genera, but share common tertiary structures. Depending on the retrovirus, Gag polyproteins may contain additional domains, e.g., those needed for release from the plasma membrane or during early replication. In solution, HIV-1 Gag is in a monomer–dimer equilibrium, whereas MLV Gag is monomeric (12). Gag binds the plasma membrane through its MA domain and recruits the viral RNA genome through its NC domain, leading to Gag oligomerization. The CA domain and immediate downstream residues mediate protein interactions in the immature shell, and, following proteolytic cleavage, CA forms the mature viral capsid. Proteolytic maturation is accomplished by the viral protease (PR). PR cleaves Gag in three to eight positions, depending on the genus, which leads to separation of individual domains and triggers a dramatic morphological rearrangement of the viral core, which is essential for viral infectivity (3, 13).

The morphology of the immature Gag shell appears spherical on thin-section EM for all orthoretroviruses studied to date. In contrast, the mature cores appear quite different depending on the viral genus. Lentiviruses including HIV-1 contain cone-shaped cores. This cone exhibits fullerene geometry with approximately 200 CA hexamers and 12 CA pentamers (five and seven at the narrow and wide ends, respectively) forming the protein lattice (7, 14, 15). Other retroviruses, such as RSV and MLV, display polyhedral or nearly spherical capsid cores, which are believed to exhibit fullerene geometry as well, but with different positioning of the 12 CA pentamers (16, 17). Furthermore, mature capsids may be polymorphic even within the same retrovirus genus. Higher resolution structural information on authentic mature retroviral capsids is currently only available for the cone-shaped HIV-1 core (7).

Compared with HIV-1 Gag, MLV Gag lacks the C-terminal p6 domain and the two spacer peptides (SPs) separating HIV-1 CA and NC (SP1) and NC and p6 (SP2) (18). On the contrary, MLV Gag contains an additional p12 domain between MA and CA, which is important for virus formation and during early replication stages (19, 20). The SP1 domain of HIV-1 Gag lies downstream of CA and forms a stable six-helix bundle, with the CA-SP1 cleavage site being part of this helix (5, 21). Processing of this site occurs late during virion maturation and is essential for the formation of the mature cone-shaped capsid inside the virion. MLV Gag contains a highly charged region toward the C-terminal end of its CA domain, which has been shown to adopt a helical conformation in vitro based on secondary structure prediction (22) and NMR analysis (23), and which is critical in assembly (22, 24). Therefore, this region has been termed the charged assembly helix (CAH) and has been suggested to fulfill a similar function as HIV-1 SP1 during maturation, while not being proteolytically cleaved. However, structural information on MLV CA is currently only available for the N-terminal domain (CA-NTD), for which the crystal structure of a hexamer has been solved (25). No structure of the MLV CA C-terminal domain (CA-CTD) has been determined to date, and the detailed protein interactions in the immature and mature CA lattice of MLV are not known.

In this study, we have determined the structure of the truncated MLV CA-CTD by X-ray crystallography and have resolved the architecture and CA lattice structures of immature and mature MLV particles by cryo-ET and subtomogram averaging. By comparison with the CA structures from HIV-1 and retroviruses from other genera, these data revealed conserved and variable features of retroviral maturation, and thereby allow general principles of retrovirus assembly and maturation to be deduced.

## Results

### Crystal Structure of MLV CA-CTD.

**Fig. 1.**
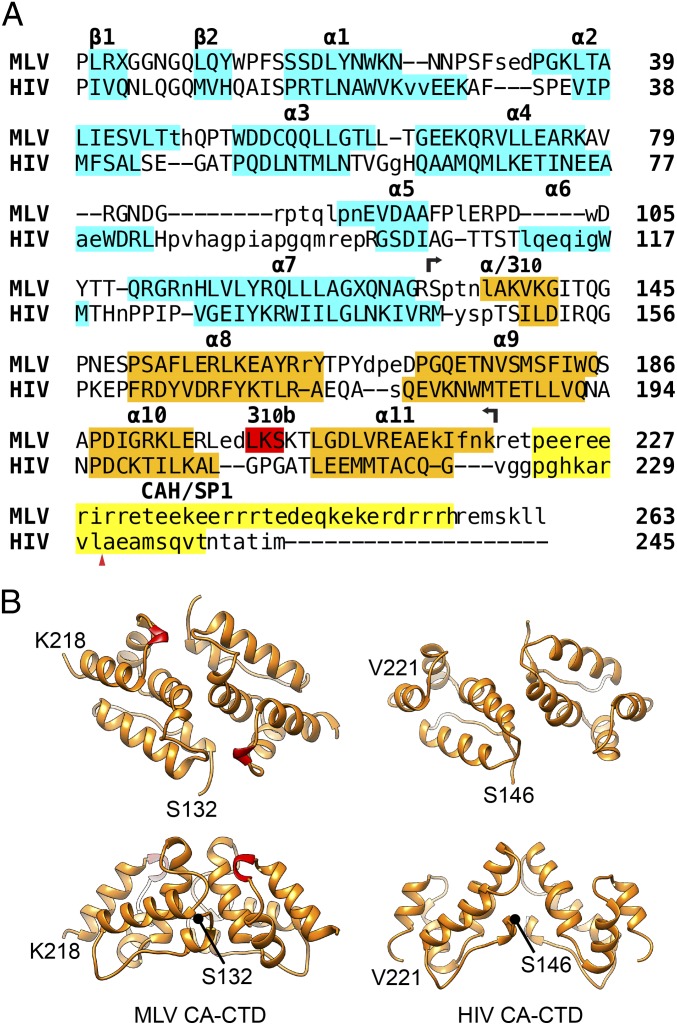
(*A*) Structure-based sequence alignment of MLV CA and HIV CA-SP1. Structurally equivalent residues are in uppercase; structurally nonequivalent residues are in lowercase. Secondary structure elements in CA-NTD [MLV, PDB ID code 1U7K (25); HIV, PDB ID code 5HGK (32)] and CA-CTD [MLV, present study; HIV, PDB ID code 5L93 (5)] are highlighted in cyan and orange, respectively. MLV CAH and HIV downstream CA-CTD-SP1 helix are yellow. The additional helical turn (3_10_b) found in MLV CA-CTD is colored red. The extent of the MLV CA-CTDΔCAH (residues 132–218) construct that was crystallized is indicated by two black arrows. The red triangle denotes the PR cleavage site between HIV CA and SP1. Note that MLV has an α-helix at the position of the 3_10_ helix between helices 7 and 8 in HIV. (*B*) Cartoon representations of the crystal structure of MLV CA-CTDΔCAH dimer described here. The mature HIV CTD dimer for comparison [residues 146–221; PDB ID code 4XFX (8)]. 3_10_b is colored in red.

A detailed view on the protein interactions of the immature and mature MLV CA lattice is best achieved by fitting structural models of the two independently folded CA domains determined by X-ray crystallography or NMR spectroscopy into densities for the assembled lattice obtained by cryo-ET and subtomogram averaging. For this purpose, we sought to obtain a high-resolution structure of the MLV CA-CTD. We initially expressed residues 132–263 (Fig. 1*A*) of MLV CA in *Escherichia coli* and purified the resulting protein. We were, however, unable to crystallize this protein. Given that residues downstream of the globular CA-CTD are generally disordered in retroviral CA proteins (e.g., ref. 26), we decided to purify a truncated MLV CA-CTD (residues 132–218, CA-CTDΔCAH) lacking the charged residues downstream of the predicted globular domain (i.e., CAH; Fig. 1*A*). The structure of the truncated MLV CA-CTD was determined at 1.89-Å resolution by single-wavelength anomalous diffraction (SAD). The crystal belonged to the space group P3121 with one CA-CTDΔCAH dimer in the asymmetric unit. As expected, the overall fold of the MLV CA-CTD is very similar to that observed for other retroviruses (e.g., HIV-1; Fig. 1*B*). The dimerization interface includes helix 9, as previously shown for other retroviruses (16). A short helical turn was found in the MLV CA-CTD between helices 10 and 11 (Fig. 1*B*, red) that is not conserved between genera and that contributes to the dimerization interface. We refer to this helix as helix 3_10_b.

### Structure of the Immature MLV Gag Lattice.

Immature MLV particles were purified from the culture medium of HEK293T cells transfected with a proviral MLV plasmid containing a mutation in the viral PR gene. Virus particles were imaged by cryo-ET (*SI Appendix*, Fig. S1*A*) and showed characteristic features of immature retroviruses including a striated Gag layer underlying the viral membrane. A total of 79 immature virus particles were extracted from 20 reconstructed tomograms and subjected to subtomogram averaging to determine the structure of the CA layer. The virus particles had a diameter of 114 ± 5 nm, a narrower distribution than has been previously described (11, 27). Subtomogram averaging was performed essentially as described previously (4): subtomograms were extracted at positions underneath the spherical viral membrane and were subjected to iterative alignment and averaging, resulting in a structure of the hexameric immature CA lattice at 6.6-Å resolution. The structure shows two distinct curved layers corresponding to CA-NTD and CA-CTD (*SI Appendix*, Fig. S1*B*). The hexamer–hexamer distances of CA-NTD and CA-CTD hexamers were 8.0 ± 0.3 nm and 7.3 ± 0.3 nm, respectively (*SI Appendix*, Fig. S1*B*), consistent with previous measurements (11) and similar to those in other retroviruses (28, 29).

**Fig. 2.**
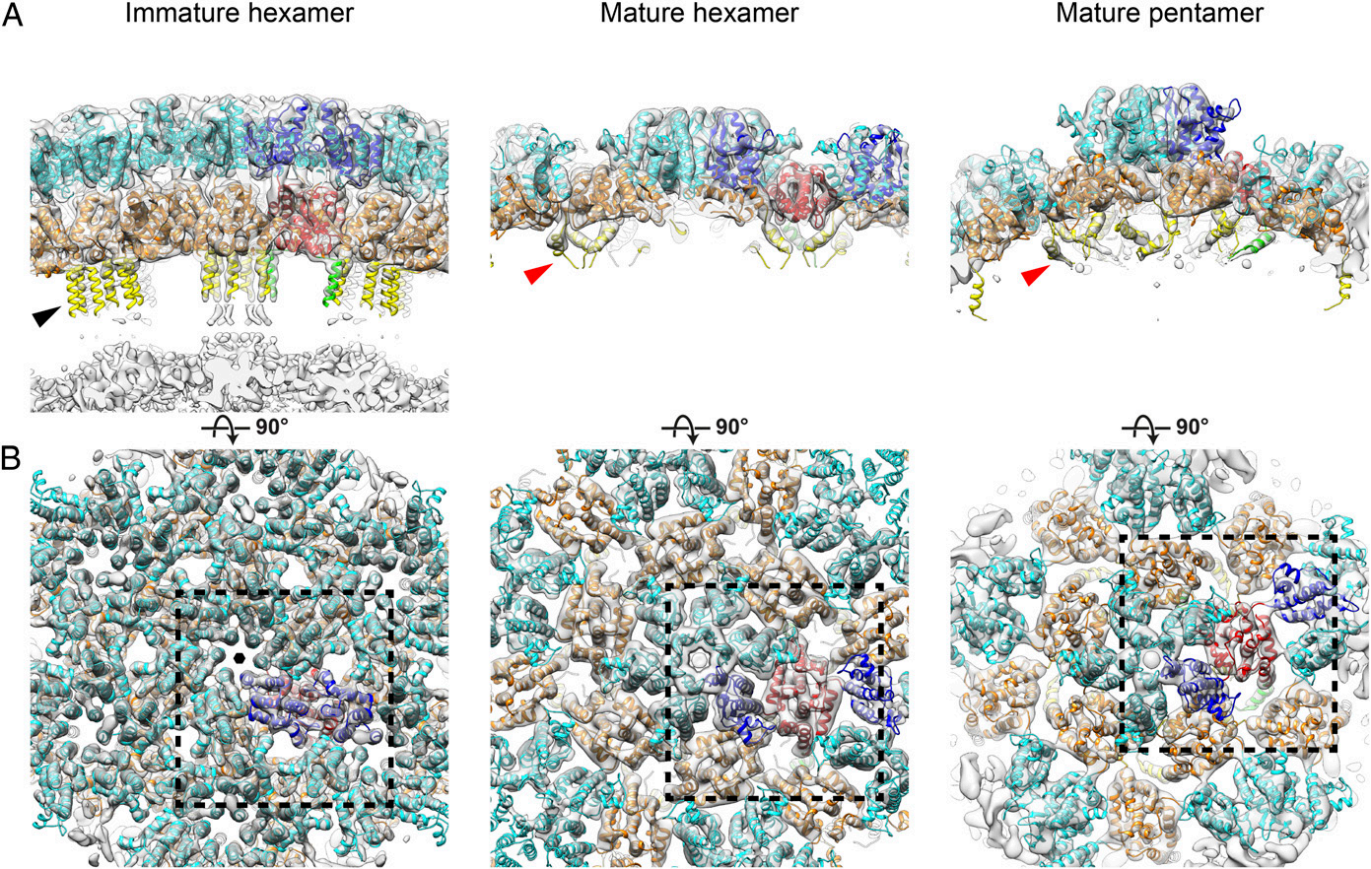
Structures of the CA region determined from immature and mature MLV virus particles by subtomogram averaging. (*A*) Cryo-ET density (gray) for CA lattice viewed tangentially. The isosurface level is set to allow fewer ordered parts of CAH and NC layer to be observed. The structural model is fitted and colored (NTD in cyan, CTD in orange, CAH in yellow). One CA dimer is highlighted by coloring the respective domains blue, red, and green. The CAH is marked by a black arrowhead (immature six-helix bundle) and a red arrowhead (mature, three helices on threefold axis). (*B*) As in *A*, but viewed from outside the Gag lattice. Black rectangles with dashed lines indicate regions shown at higher magnification in Fig. 3. The features shown in this figure are also illustrated in Movie S1.

**Fig. 3.**
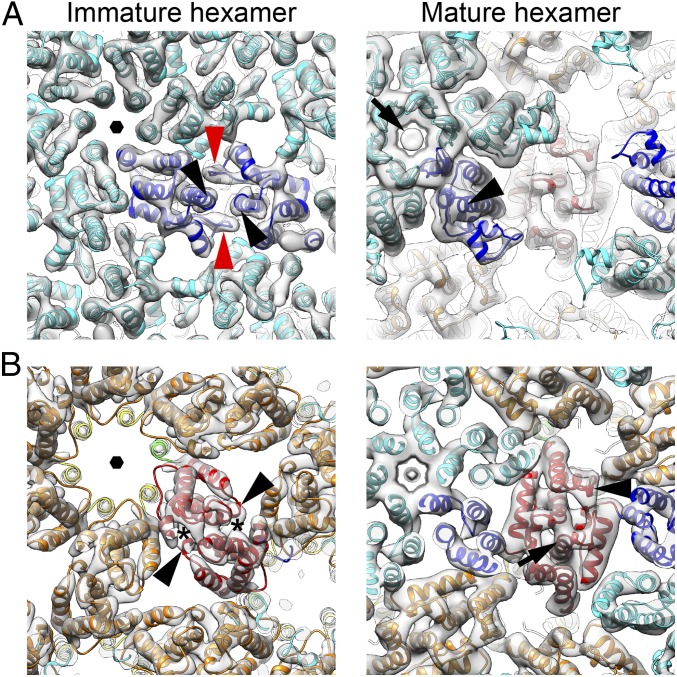
Magnified views of the CA region within the black rectangles in Fig. 2. (*A*) As in Fig. 2, but focused on CA-NTD. The ordered loop (red arrowhead) between helices 4 and 5 and the C-terminal end of helix 7 (black arrowhead) form the immature CA-NTD dimeric interface. A faint additional density (black arrow) is observed within the central pore of mature CA-NTD hexamer structure. Black hexagon indicates the sixfold axis. (*B*) As in *A*, but focused on CA-CTD. The densities marked with asterisks correspond to the immature CA-CTD dimer interface formed by 3_10_b. Black arrowhead indicates the 3_10_b helix, and black arrow indicates helix 9. The features illustrated in this figure are also shown in Movie S1, in which the reader may find the spatial relationships easier to visualize. The equivalent views of the mature pentamer are shown in *SI Appendix*, Fig. S3*D*.

To interpret the structure, we extracted a CA-NTD monomer from the available crystal structure of the reported CA-NTD hexamer [Protein Data Bank (PDB) ID code 1U7K] (25) and a CA-CTD monomer from our CA-CTDΔCAH dimer structure and fitted them as rigid bodies into the immature MLV CA cryo-ET map. Both domains fit well into the structure: all helices and loops were accommodated inside recognizable densities (Fig. 2*A*). After fitting the CA-NTD and the truncated CA-CTD into the electron density map, there was clear density projecting toward the center of the particle that was not occupied by the domain structures. This density comprised six extended rod-like densities consistent with α-helices, running parallel to the sixfold axis ∼15 Å apart (Fig. 2*A*, black arrowhead). These densities were weaker than those observed for CA-NTD and CA-CTD, suggesting that the helices may be slightly flexible. As this six-helix structure is positioned between the truncated CA-CTD and NC domains of Gag, it must consist of residues of the CAH that had been deleted in the CA-CTDΔCAH protein used for X-ray crystallography. The CA-CTD part missing in the crystal structure consists of 45 aa. Residues 222–256 have been predicted and confirmed in an in vitro assembly reaction and by NMR spectroscopy to form an α-helix (i.e., the CAH) that would be ∼52 Å long (22, 23). The distance between the base of the CA-CTD and the ribonucleoprotein (RNP) layer is ∼40 Å; however, clear rod-like densities cover only the first 23 Å of this distance in the sharpened map (Fig. 2*A*). To determine whether the helical density is longer but not visible at high resolution as a result of flexibility relative to CA-CTD, we realigned the data by using a mask including the base of CA, RNP, and the region between them. In the realigned data, the rod-like densities extend into the RNP layer and are ∼50 Å long (*SI Appendix*, Fig. S1*C*), consistent with the predicted length (23). At the resolution of our structure, we cannot perform de novo determination of the CAH structure in the six-helix bundle, but have modeled the N-terminal 15 residues of the CAH (P222–K236) as a six-helix bundle of this length and placed it into the determined electron density (Fig. 2*A*, black arrowhead, and Fig. 3*B*, black hexagon). Based on the length of the rod-like density, it is likely that at least 18 further amino acids of the CAH adopt a helical conformation, giving a total of at least 33, again consistent with in vitro observations (23). The remaining residues are buried within the RNP density layer, and we cannot address their conformation.

In the CA-CTD, an additional density is seen for helix 3_10_b in the subtomogram averaged structure (Fig. 3*B*, black arrowhead), indicating that the helix is present in the immature virus and is not an artifact of crystallization. The CA-CTD dimerization interface involves interaction between helices 9, as is the case for other immature retroviruses, but also seems to include an interaction between helix 3_10_b and the base of helix 9 in the vicinity of W184 (Q192 in HIV-1; Fig. 3*B*, asterisk).

The CA-NTD forms a hexamer in the immature lattice, at the center of which is a parallel helical bundle formed by helix 1, surrounding a central pore (Figs. 2*B* and 3*A*, black hexagon). Hexamers are packed together by a dimeric interface in which the C-terminal end of helix 7 (Fig. 3*A*, black arrowhead) interacts with the loop between helices 4 and 5 (Fig. 3*A*, red arrowhead). This loop is clearly visible in the EM density (Fig. 3*A*, red arrowhead), suggesting that it is held in a rigid conformation by these interactions. Amino acid substitutions in the loop between helices 4 and 5 have caused defects in virus assembly (30, 31), consistent with a role of the loop in mediating CA–CA interactions in the immature virus. At the equivalent position in the sequence of the HIV-1 CA domain is the extended cyclophilin binding loop. The cyclophilin binding loop of HIV-1 does not, however, contribute to hexamer packing, and is not well ordered in the immature virus (5).

### Structure of the Mature MLV CA Lattice.

Purified mature, infectious MLV particles from the culture medium of transfected HEK293T cells were inactivated and subjected to cryo-ET, and 134 mature viruses were identified in 65 tomograms (*SI Appendix*, Fig. S1*A*). The virus particles had a diameter of 114 ± 6 nm (*n* = 66), similar to that of the immature virus preparation.

We applied subtomogram averaging to obtain a density map of the CA hexamer in the core of the mature virions at 7.2-Å resolution. As previously described for HIV-1, the mature MLV CA lattice is thinner than the immature Gag shell, consisting of a single layer including CA-NTD and CA-CTD (Fig. 2*A*). The hexamer–hexamer spacing in the middle of the mature CA layer was 10.0 ± 0.4 nm (*SI Appendix*, Fig. S1*B*), consistent with previous measurements from HIV-1 (28), and larger than that in the immature virus.

As for the immature virus, we fitted monomers of CA-NTD and CA-CTDΔCAH from the crystal structures into the mature CA cryo-EM map as rigid bodies to generate a model for the mature lattice (Fig. 2*A*). All helices were identified, and loops could also be observed at lower isosurface levels. We compared the arrangement of the monomers in our mature lattice model with the monomer arrangements in the crystal structures of the CA-NTD hexamer (25) and the CA-CTD dimer (present study), respectively. The structures were essentially identical (*SI Appendix*, Fig. S2), indicating that the oligomeric forms present in the crystal structures are representative of the arrangement in the mature virus.

The observed density extends beyond the C terminus of the truncated protein used for crystallography in a short rod-shaped density toward the center of the core (Fig. 2*A*, red arrowhead). This density would accommodate an α-helix corresponding approximately to residues P222–R230 of the CAH. Three of these short helices cluster around the threefold symmetry axis, bringing their C-terminal ends toward one another (Fig. 2*A*, red arrowhead, and *SI Appendix*, Fig. S3 *A* and *B*, triangle). Ordered density is not seen at the equivalent position in mature HIV-1, and no well-ordered density was observed for the remaining part of the CAH in the mature MLV CA hexamer. In the absence of ordered density for downstream regions in immature or mature MLV, we were unable to further interpret previous mutagenesis data in this region.

The CA-CTD interacts at the twofold axes via helix 9 (Fig. 3*B*, black arrow), as seen in other retroviruses, but also via interactions between K201 in helix 3_10_b and D171 in the loop between helices 8 and 9 (Fig. 3*B*, black arrowhead). Helix 3_10_b therefore contributes to the dimerization interface in immature and mature virus particles, but via interaction with different partners.

As for HIV-1, in mature MLV, there is a CA-CTD–CA-NTD interface. This involves helix 4 and helix 8 (*SI Appendix*, Fig. S3 *A* and *C*, asterisk). The CA-NTD hexamer, as previously described based on the crystal structure, centers around a tilted helical bundle formed by helix 1 (Figs. 2*B* and 3*A*). Above this bundle, the predicted β-hairpin is visible. Together, helix 1 and the β-hairpin form an extended pore through the hexamer. A faint additional density was seen within the central pore of the mature CA-NTD hexamer structure that could represent a negatively charged ion (Fig. 3*A*, black arrow) (7, 32). This density is in a position where it could be coordinated by a ring of six arginines within the β-hairpin.

Following the procedure described by Mattei et al. (7), we analyzed the distribution of hexamers in the CA lattices, searching for pentamerically coordinated positions. We identified 1,299 pentamerically coordinated positions from the 134 virions in our dataset, extracted subtomograms at these positions, and iteratively aligned and averaged them to generate a structure of the mature MLV CA pentamer at a resolution of 8.6 Å. CA domains were fitted into the map as rigid bodies. The CA-CTD layer displays high curvature at the pentamer positions, and the CA-NTD pentamer protrudes further outward from the CA-CTD layer than in the hexamer (Fig. 2*A*). Comparison of the interfaces between neighboring CA molecules in the pentamer and hexamer revealed subtle differences in these interactions but no dramatic opening of the interdomain cleft as we previously observed in the HIV-1 pentamer (7). The MLV CA-NTD pentamer is more tightly packed around the fivefold symmetry axis than the hexamer, resulting in an even narrower pore in the center and higher local positively charged electric potential contributed by arginines in the β-hairpin (Figs. 2*B* and 3*A* and *SI Appendix*, Fig. S3*D*).

### Architecture of the MLV Gag and CA Lattices.

**Fig. 4.**
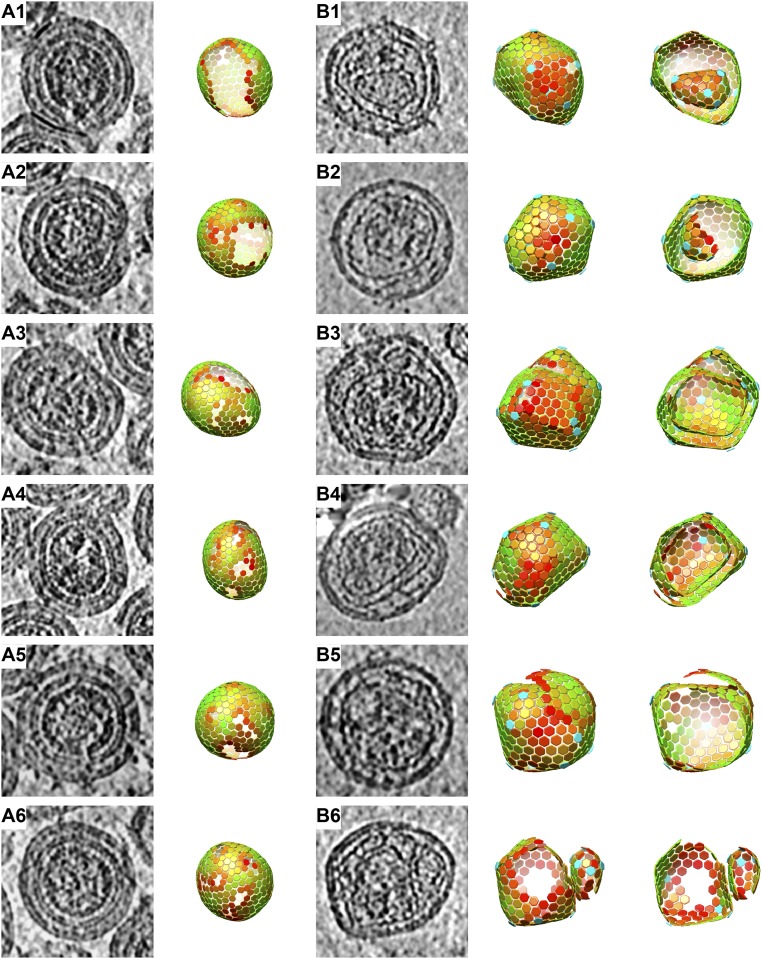
Morphologies of immature and mature MLV particles. Slices through tomographic reconstructions of particles (*A* and *B*, *Left*) and lattice maps derived from subtomogram averaging that illustrate the positions of CA hexamers (*A* and *B*, *Right*) are shown for six representative immature (*A*) and mature viruses (*B*). For the mature virus, a “cut-open” view of the lattice map is also shown. Colors of hexagons denote the CCC of alignment on a scale from low (red) to high (green). The CCC range in each lattice map has been set between the minimum and the maximum CCC value present in the virus. *B1* and *B2* contain nested cores in which the inner core is complete or incomplete. *B3* and *B4* contain cores with spiral morphology. *B5* contains a complete polyhedral core with an isolated sheet of CA lattice outside. *B6* contains two separate cores. Perpendicular views of the same mature viruses are shown in *SI Appendix*, Fig. S4. Further examples of mature cores are provided in *SI Appendix*, Fig. S5.

By applying subtomogram averaging, the positions and orientations of CA hexamers within the respective lattices can be identified, and the architecture of the Gag or CA shell can be visualized by placing hexamers in a 3D volume to represent the overall arrangement of the lattice. We first performed this analysis for the immature lattice (Fig. 4*A*). All 79 immature Gag shells we observed were approximately spherical but incomplete. As previously reported for HIV-1 (4, 33), each immature Gag lattice contained one larger defect and smaller defects with various sizes. Some defects are surrounded by five CA hexamers and are pentamerically coordinated, but no structural density at these positions was resolved by using subtomogram averaging, suggesting that they are pentameric holes rather than CA pentamers.

In the tomograms of mature virions, we observed a variety of core morphologies, including open, multiple, and multilayered cores (Fig. 4*B*). Some of these morphologies are visible in electron micrographs published in previous studies (11, 27, 34), but have not been further analyzed to our knowledge so far. The mature CA lattice is smoother than the immature Gag lattice, and there is therefore a higher number of false-positive and -negative identifications of hexamers for mature than immature viruses, hindering analysis of the lattice architecture. For a subset of the data (66 viruses from 33 tomograms with high defocus values), we further processed the particles to minimize false-positive and false-negative findings and visualized the arrangement of pentamers and hexamers to study the architecture of the mature viral cores (Fig. 4*B*).

The mature MLV cores consisted of flat areas of lattice joined at seams with locally higher curvature, and with pentamers positioned at the highly curved vertices. Some virus particles (38 of 66) contained spiral or nested polyhedral cores (Fig. 4 *B3* and *B4* and Movies S2 and S3), and other particles (27 of 66) contained polyhedral cores together with isolated sheets of CA lattice (Fig. 4*B5*). One of the 66 particles contained 2 separate cores of very different sizes (Fig. 4*B6*). In only a minority of these virus particles (5 of 66) was one of the polyhedral cores clearly closed (Fig. 4 *B1* and *B2*). *SI Appendix*, Fig. S5 shows more examples of typical morphologies of mature MLV cores. Within spiral or nested cores, more than 12 pentamers are required to accommodate the increased total curvature, and we observed as many as 24 pentamers within a single virus particle (Fig. 4*B1*).

**Fig. 5.**
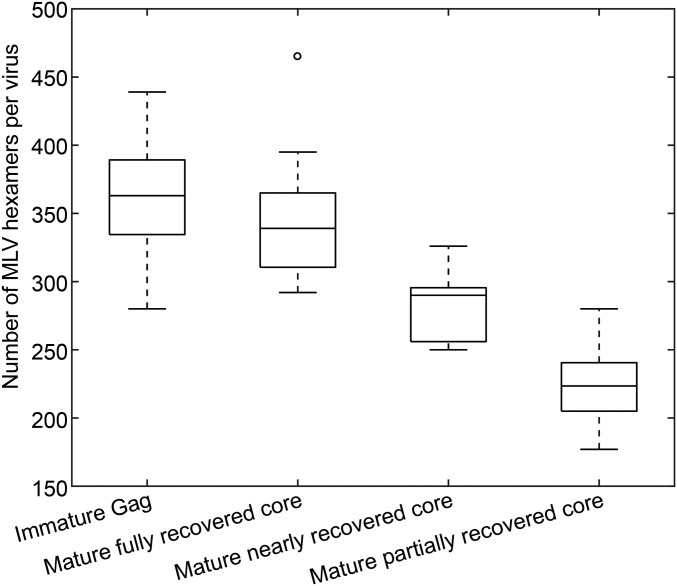
The number of CA hexamers in the lattice map measured per virus in immature and mature MLV. For mature MLV, separate numbers are given for virus particles according to whether the lattice is partially, nearly, or fully recovered during image analysis. The degree of recovery is derived by assessing what fraction of the CA lattice seen in the tomographic reconstruction of the particle was marked and aligned in lattice maps. Immature virus particles are all thought to be fully recovered. The averages are 363 ± 36 (immature), 344 ± 44 (mature fully recovered), 282 ± 23 (mature nearly recovered), and 223 ± 27 (mature partially recovered), respectively, suggesting that the numbers of CA hexamers in immature MLV and mature MLV (if fully recovered) are similar.

To determine the assembly mode, we wished to count the number of CA hexamers contributing to the mature core. The irregular morphology of the cores makes it challenging to generate complete maps of hexamers and pentamers in all cases (*Materials and Methods*), and, in many cases, patches of lattice were visible in tomograms that are not represented in lattice maps. We therefore sorted the lattice maps into three classes based on how completely the respective lattice map represents the lattice densities that are visible in the tomogram: fully, nearly (in which small portions of lattice that are visible in the tomogram are not represented by the lattice map), and partially (in which substantial regions of lattice that are visible in the tomograms are not represented by the lattice maps). We counted the total number of mature CA hexamers in each class (Fig. 5), and compared it vs. the number of hexamers observed in the immature virus particles. The results suggest that the number of CA hexamers in the complete mature lattice is approximately the same as the number of Gag hexamers in the immature virus, suggesting that most CA molecules assemble into the mature CA lattice.

## Discussion

High-resolution structural analysis of retroviral CA domains from different genera has shown that secondary structure elements and tertiary structures are largely conserved, but they differ strongly in sequence. Surprisingly, the quaternary structural arrangements of CA domains within the immature lattice again differ significantly among genera: although the arrangement of the CA-CTD appears mostly conserved among RSV, M-PMV, and HIV-1, the CA-NTD arrangements are quite different. The mature quaternary arrangement has previously been determined within a virus particle only for HIV-1. Here, by combining our crystal structure of the MLV CA-CTD with EM structures of the CA lattice determined within intact virions, we have been able to build structural models for immature and mature states of the MLV CA lattice. We discuss the implications of these models for the arrangement and interactions of CA within the immature and mature lattices, their implications for structural conversion during maturation, and finally regarding the overall architecture of the immature and mature protein shell.

The arrangement of CA within the mature MLV CA lattice is remarkably similar to that in the mature HIV-1 capsid, whereas the overall architecture and morphology of these two capsids appears completely different (as discussed later). Both CA proteins assemble into hexamers and pentamers, and the quaternary structures of these oligomers are very similar. The arrangement of CA-CTD in the immature MLV lattice is generally similar to that previously reported for immature M-PMV and HIV-1, with dimerization mediated by helix 9. In MLV, the dimer interface appears to be further stabilized by interactions involving the novel helix 3_10_b. In contrast to the conserved immature CA-CTD arrangement, the immature MLV CA-NTD interfaces diverge strongly from any of the other three immature lattices previously described. The immature MLV CA-NTD arrangement is more similar to that seen in mature HIV-1 and MLV lattices: in all cases, helices 1 and 2 are located toward the center of the sixfold axes and form a large helical bundle. The contrast between the conserved immature CA-CTD arrangement and the divergent immature CA-NTD arrangement (*SI Appendix*, Fig. S6) suggests that the major protein interactions driving immature retrovirus assembly are mediated by CA-CTD. The immature CA-NTD arrangement may be free to diverge because this CA domain performs its main, conserved functions only after maturation, when it promotes mature core assembly and mediates the interactions with host cell factors that occur when the mature core has entered a new target cell.

In HIV-1, immature and mature lattices differ by a rotation of the CA-CTD dimer around the helix 9 interface and by a complete inversion of the NTD repositioning helix 1 from the interhexamer dimer interface into the helical bundle in the center of the hexamer (4). This transition requires the breaking of almost all of the interactions in the immature lattice. In contrast, in MLV, helix 1 is present at the center of the hexamer in immature and mature viruses, and the CA-NTD hexamer structures can be interchanged by a slight tilt of the CA-NTD toward the hexameric axis, bringing the newly formed β-hairpin into the center and narrowing the pore (Movie S4). The immature interhexamer interactions formed by the loop between helices 4 and 5 in the CA-NTD are broken, removing all CA-NTD hexamer–hexamer interfaces and exposing the C-terminal end of helix 7 (residues 110–117) on the outside of the hexamer (Fig. 3*A*, black arrowhead). These residues are involved in defining the tropism of MLV for the restriction factor Fv1 (35) and therefore are accessible for binding in the assembled mature core. During maturation, the CA-CTD dimer undergoes a twist around the helix-9 interface, which moves the part of the CAH still ordered in the mature lattice from the sixfold symmetry axis to the threefold symmetry axis. Meanwhile, helix 3_10_b transitions from interacting with residues in the 7–8 linker or the C terminus of helix 9 to forming an electrostatic bond with an aspartate in the loop between helix 8 and 9 (Movie S4).

The similarities between the immature and mature CA lattices in MLV contrast with the differences seen in HIV-1. The immature MLV lattice can be converted into a mature lattice by rotations around existing protein–protein interactions (Movie S4), and it is tempting to imagine that structural maturation is achieved by a smooth expansion of the lattice. Expansion, however, is constrained by the viral envelope. For HIV-1 and MLV, there are dramatic architectural changes at maturation that require closure of gaps in the CA lattice, formation of pentameric vertices, breaking of existing CA–CA interactions, and formation of new CA–CA interactions. Indeed, in MLV, there is often a transition from a single CA lattice in the immature virus to two separate CA lattices in the mature virus. These architectural changes are inconsistent with an exclusively displacive transition (36). We previously proposed that the structural transition in HIV-1 could be achieved by a disassembly of the immature lattice into monomers or dimers, or by a “molecular dance” in which new, mature CA–NTD interactions stabilized hexamers or other higher-order oligomers during rearrangement of the CA-CTD, in both cases followed by reassembly (4). Ning et al. (37) have suggested a similar model: a displacive transition of a patch of the lattice followed by a disassembly/reassembly event. For MLV, the similarity between the immature and mature CA-NTD hexamer structures, in both cases involving helix 9 in the CA-CTD and helices 1 and 2 in the CA-NTD (Movie S4), means that the molecular dance would be more subtle: only small changes in the CA-NTD layer would be required to stabilize the mature hexameric interactions. Nevertheless, the constraints of core formation are similar to those in HIV-1: disassembly and reassembly are required for mature core growth, which could initiate from small oligomers or from one or more patches of mature lattice themselves formed via a local molecular dance displacive transition.

In immature HIV-1 particles, the C-terminal end of CA and the adjacent SP1 domain form a six-helix bundle. This helical bundle must unfold for structural maturation of the CA lattice. Here, we show that, in immature MLV, the CAH at the C-terminal end of CA forms a similar helical bundle. In HIV-1, disassembly of the helical bundle is followed by proteolytic cleavage at a site between CA and SP1. In contrast, no cleavage occurs in this region in the case of MLV. Nevertheless, our analysis of the mature MLV lattice reveals that, upon maturation, similar to HIV-1, the six-helix bundle disassembles and the helical arrangement of the CAH is largely lost. This is consistent with HIV-1, in which disassembly of the helix, but not proteolytic cleavage, is the essential step for structural maturation (38).

The general architecture of the immature MLV lattice is similar to the previously determined HIV-1 and M-PMV Gag shells: it consists of a hexameric arrangement of Gag in which curvature is accommodated by the presence of multiple small defects and one larger defect rather than by Gag pentamers. Accordingly, this seems to be a general feature of retrovirus formation: a curved hexameric lattice is formed by assembly of the complete Gag polyproteins and irregular defects accommodate curvature strain. The large gap in the lattice is likely a result of Gag-mediated recruitment of the cellular ESCRT complex, whose assembly at the bud neck appears to terminate Gag assembly and mediates virion release from the cell surface (39).

The global architecture of the CA lattice in the mature MLV is different than those in other retroviruses. It is generally thought that retroviral cores, when assembled properly, form closed fullerene structures in which 12 pentamers accommodate curvature in the hexameric lattice. In this model, the positions of the pentamers determine the shape of the core and differ in different retroviruses. We have observed that, within HIV-1 particles, even though many cores contain gaps and defects, a subset of cores does indeed form perfect closed fullerenes (7). Cryo-ET observations of RSV (40) and Human T-cell leukemia virus-1 (41) are also consistent with the presence of a single, pleomorphic, closed fullerene core in a significant fraction of particles. The cores we observed in MLV were, however, mostly multilayered or spiral structures. In the rare cases in which a complete core was assembled, other partial cores were present in addition. Mature MLV cores have been previously described to have irregular polygonal shapes (11), but examination of cryo-EM images of mature MLV in the literature (11, 27, 34) suggests that they have multilayered or multiple cores, consistent with the observations here. This appears to be independent of whether virus particles were produced in rodent (11, 27) or primate cell lines (11, 34).

When the fullerene HIV-1 core has been assembled, the large fraction of remaining CA protein (∼50% of all virion-associated CA) is not required and is presumably elsewhere in the viral particle (28, 42). In RSV, the fraction of CA incorporated into the mature core depends on core morphology but is largest in angular cores, where it is ∼80% (40). In contrast, in MLV, we observed that most of the CA protein assembles into core-like structures. This implies that the critical concentration for assembly of MLV CA is lower than that for assembly of RSV or HIV-1 CA. As the mature CA lattice is less densely packed than the immature CA lattice (10 nm vs. 8 nm), a single-layered mature polygonal core incorporating most or all CA proteins would be larger than the immature CA layer and could not fit within the viral membrane. Accordingly, we suggest that mature MLV always contains multilayered or multiple cores, and no subpopulation with a single closed core exists. This is in marked difference to HIV-1, in which single, closed cores can be observed (7).

The mode of mature MLV core assembly appears to be inherently irregular: CA hexamers assemble a curved lattice, incorporating pentamers in positions of high curvature; this process continues until most CA molecules inside the virion have polymerized into the lattice, leading to multiple capsids, spiral capsids, and other incompletely closed structures. As the total curvature of a multilayered structure is greater than that in a sphere, more than 12 pentamers are present in MLV particles. The presence of pentamers, rather than gaps as in the immature lattice, suggests that having a CA lattice without holes may be important during early stages of the MLV lifecycle. Although we cannot rule out that the few completely closed MLV cores constitute the infectious population, we prefer to speculate that the wrapping of the viral RNP by CA is sufficient for function, even if the core is not a closed polyhedron.

## Materials and Methods

### Protein Production and Purification.

Detailed production and purification of MLV CA-CTD (residues 132–263) and CA-CTDΔCAH (residues 132–218) were described previously (23). The proteins were produced in *E. coli* BL21(DE3) RIL cells. The cells were harvested and lysed, and the soluble fraction was precipitated with ammonium sulfate. The precipitate was recovered by centrifugation, resuspended, and dialyzed against phosphate buffer (10 mM Na_2_HPO_4_, pH 6.0, 10 mM NaCl). The proteins were then purified by ion-exchange chromatography (HiTrap SP XL) and size-exclusion chromatography (50 mM Tris, pH 7.0, 100 mM NaCl; HiLoad 16/600 Superdex 75 pg). The purified proteins were concentrated to 40 mg/mL (MLV CA-CTD) or 37 mg/mL (CA-CTDΔCAH), flash-frozen in liquid nitrogen, and stored at −80 °C.

### Crystallization and Structure Solution.

Conditions for MLV CA-CTDΔCAH crystallization were screened at the European Molecular Biology Laboratory (EMBL) Heidelberg Crystallization Platform by using commercial (Hampton Research, Molecular Dimensions, and Qiagen) and in-house crystallization screening kits. Protein crystals grew at 20 °C in sitting drops containing 37 mg/mL CA-CTDΔCAH, 4% PEG 3350, 0.1 M Hepes, pH 8.2, 5 mM CoCl_2_, 5 mM CdCl_2_, 5 mM MgCl_2_ and 5 mM NiCl_2_. Crystals were harvested manually and flash-frozen in liquid nitrogen with 30%vol/vol glycerol as a cryoprotectant. The X-ray diffraction experiment was conducted on a rotating-anode diffractometer at EMBL Heidelberg. The data set containing 180 diffraction patterns was reduced by using XDS (43), and a significant anomalous signal was indicated. As the Cu K⍺ X-ray wavelength (1.5418 Å) is compatible with the absorption edge of cobalt (1.6083 Å), we attempted to solve the structure by SAD with cobalt atoms. The screw axis in the crystal was determined in POINTLESS (44), resulting in the correct space group of P3_1_21. The anomalous differences, substructure solution, phasing, and density modification were implemented by using SHELX C/D/E (45) in HKL2MAP (46). One cobalt atom site was located in the asymmetric unit with correlation coefficients (CC and CCweak) of 47.10 and 22.40. The initial model was built in CCP4 ARP/wARP (47, 48). Iterative model refinements were carried out by using COOT (49) and PHENIX (50). The refined structure was validated by using MolProbity (51). All details of data processing and refinement are listed in *SI Appendix*, Table S1.

### Virus Production and Purification.

HEK293T cells were seeded in 175-cm^2^ flasks (eight flasks per particle preparation). On the following day, cells were transfected with 75 µg plasmid DNA per flask by using a standard CaPO_4_ transfection procedure. For production of immature and mature MLV particles, the proviral plasmids used were M2204 (52), containing a D32L mutation in PR, and the infectious clone pRR390 (53), respectively. At 48 h post transfection, tissue culture supernatants were harvested and, in the case of WT virus, checked for infectivity. Culture media were passed through a 0.45-µm nitrocellulose filter and concentrated by ultracentrifugation through a 20% (wt/wt) sucrose cushion (2 h at 28,000 rpm in a Beckman SW32 rotor at 4 °C; Beckman Coulter Life Sciences). Pellets were resuspended in PBS solution and further purified by ultracentrifugation through an iodixanol gradient as described previously for HIV-1 (54). The clearly visible virus containing fraction was collected, diluted 1:10 with PBS solution, and concentrated by ultracentrifugation (45 min at 44,000 rpm in a Beckman SW60 rotor, 4 °C). Pellets were gently resuspended in PBS solution. Samples were fixed with 1% paraformaldehyde (1 h on ice) and directly prepared for electron tomography or stored in aliquots at −80 °C. Purity of samples and virus amounts were assessed by SDS/PAGE and silver staining (*SI Appendix*, Fig. S7).

### Cryo-ET.

Grid preparation and data acquisition were performed similarly for immature M-PMV and immature and mature MLV samples (*SI Appendix*, Table S2). Purified virus solution was diluted 1:1 with PBS solution containing 10 nm colloidal gold. Then, 2.5 μL of the mixture was plunge-frozen on glow-discharged C-Flat 2/1 3C grids (Protochips) by using an FEI Vitrobot Mark II. Grids were loaded into an FEI Titan Krios transmission electron microscope operated at 300 kV and imaged by using a Gatan Quantum K2 Summit direct electron detector in superresolution counting mode. Tomographic data collection was controlled by SerialEM software (55) by using a dose-symmetric tilt scheme with a 3° angular increment and a tilt range of ±60 or ±66° (56). The nominal magnification was 105,000×, giving a pixel size of 1.35 Å on the specimen. Data acquisition conditions are summarized in *SI Appendix*, Table S2.

Image preprocessing was carried out identically for all three data sets (*SI Appendix*, Table S2). All superresolution tilt image frames were drift-corrected and Fourier-cropped by MotionCor (57). Each summed tilt image before dose-filtering was used to estimate the contrast transfer function (CTF) as described previously (58). Exposure filtering was implemented according to the cumulative dose per tilt as described previously (5, 59). Motion-corrected and dose-filtered tilt series were CTF-corrected by phase flipping, and tomograms were reconstructed in IMOD (60).

### Subtomogram Averaging.

Subtomogram averaging was performed in the same way for immature MLV and M-PMV by using scripts derived from TOM (61), AV3 (62), and Dynamo (63) packages, essentially as described in ref. 58. The initial reference-free reconstruction was performed by using fourfold binned data (bin4) from one tomogram, applying a 40-Å low-pass filter with no symmetry applied, followed by two iterations with sixfold symmetry applied. Using this reference, all data were aligned at bin4 by using a 40-Å low-pass filter, and subtomograms with low cross-CCs (CCCs) or at duplicate positions in the CA lattice were removed. The dataset was then split into two half-datasets that were further processed independently. Three iterations at bin2 and two iterations at bin1 were performed, with an adaptive low-pass filter and decreasing angular search range while monitoring the resolution by using the Fourier shell correlation (FSC). After convergence, the final resolution was reported at 0.143 cutoff in phase randomization-corrected FSC curves between two masked half maps (*SI Appendix*, Fig. S8). The mask included only the central hexamer and excluded the less ordered CAH and NC. The two half maps were merged and sharpened by using a B factor measured by the Guinier plot in Relion (64).

The alignment used a mask including CA-NTD and CA-CTD layers. To better resolve the CAH densities in immature and mature viruses, we performed two alignment iterations at bin2 by using masks that included the base of CA-CTD, the presumed region of CAH, and the RNP (RNP in immature virus only). The structures after alignment were sharpened and are shown in *SI Appendix*, Fig. S1*C*.

To extract subtomograms along the surface of mature MLV cores, the surface of spiral or nested cores was manually picked on the CA layer in the tomogram and interpolated in 3D. Initial coordinates and Euler angles of mature CA hexamers were computed on this surface. After an initial alignment at bin4, the positions of the aligned subtomograms were combined with the initial manual picking of the surface to reextract the subtomograms. Subtomogram averaging procedures were then performed as described earlier to solve the mature hexamer structure and refine the lattice packing. The locations of mature CA pentamers were calculated by analyzing the pairwise distances between neighboring hexamers as described previously (7). Subtomograms were extracted from these predicted positions, averaged, and further aligned. Pentameric defects in the immature MLV CA lattice were also extracted and averaged, but no featured density was observed at these positions.

To validate the mature CA lattices structure and architecture, we repeated transfection and particle purification and collected 25 tomograms containing 77 viruses from this new preparation. Data collection and image processing were performed with similar conditions and parameters, giving a CA structure at 8.1 Å. The structure is the same as that obtained from the original preparation to the determined resolution. Core morphologies were also similar to those observed in the original preparation, including spiral and nest polyhedral cores.

### Immature M-PMV Structure.

We previously determined the structure of the CA layer within immature M-PMV particles to a resolution of 9.7 Å (4). We produced immature M-PMV particles by using the proviral plasmid pSHRM15 D26N as previously described (4). We repeated the structure determination by using the experimental conditions described earlier and obtained a structure at 7.2 Å. We flexibly fitted the previously published structural model into this higher-resolution map. The improved structural model showed better-resolved loops compared with the previously published model; it is illustrated in *SI Appendix*, Fig. S6.

### Structure Fitting and Lattice Map Visualization.

The structures of monomers of MLV CA-NTD (PDB ID code 1U7K) and CA-CTDΔCAH (present study) were fitted into the immature and mature MLV EM maps as rigid bodies by using UCSF Chimera (65). The downstream CAHs in immature and mature MLV CA-CTD were modeled as backbone-only α-helices in COOT and fitted into the density. The models of immature and mature MLV CA were obtained by combining the three fitted domains.

To generate the model of immature M-PMV shown in *SI Appendix*, Fig. S6, we took the model previously published in ref. 4, fitted it into the EM map in UCSF Chimera, and performed flexible fitting and model refinement in COOT and PHENIX.

To visualize the distribution of hexamers within the viral particles, as shown in Fig. 4, we implemented a plugin for UCSF Chimera, which can be downloaded from the following link (www2.mrc-lmb.cam.ac.uk/groups/briggs/resources/). This plugin has capabilities for marking and displaying points that are similar to the EMPackage (66), which we have used in previous studies. A geometric object or a down-sampled surface of the high-resolution reference is translated and rotated according to the parameters determined for each subtomogram and displayed in the same volume space as the tomogram (Movies S2 and S3), and can be colored according to the CCC (Fig. 4). We call this display a lattice map.

The distribution of hexamers determined by subtomogram averaging may contain false-positive and false-negative signals. We compared the determined distribution of hexamers with the densities observed in the reconstructed tomogram. We then manually removed the small number of hexamers that appeared to be false-positive findings resulting from misalignment, such as hexamers that were inconsistent with the local lattice arrangement, immature hexamers that were not positioned appropriately relative to the membrane, or hexamers identified within the lipid bilayer. For immature virus particles, there was excellent correspondence between the boundaries of the determined lattice and the edges of the underlying Gag density, suggesting that there are few false-positive or false-negative signals. For mature viruses, we primarily observed false-negative signals: we could see some regions of assembled CA in the tomograms that were not identified during subtomogram alignment. We then grouped the particles according to the completeness of the lattice map (Fig. 5).

### Data Availability.

The crystal and EM structures together with the fitted models and representative tomograms have been deposited in the PDB (https://www.ebi.ac.uk/pdbe/) under accession numbers 6GZA, 6HWI, 6HWW, 6HWX, and 6HWY, and in the Electron Microscopy Data Bank (www.ebi.ac.uk/pdbe/emdb) under accession numbers 0290–0293, 4419, 4421, and 4422. Accession numbers for all entries are listed in *SI Appendix*, Tables S1 and S2.

## Supplementary Material

Supplementary File

Supplementary File

Supplementary File

Supplementary File

Supplementary File
