# Supplementary material for: Structure and architecture of immature and mature murine leukemia virus capsids
Source: Proc Natl Acad Sci U S A. 2018 Nov 26;115(50):E11751–60. doi: 10.1073/pnas.1811580115 (PMC6294937; doi:10.1073/pnas.1811580115)
Supplement: Supplementary File [file pnas.1811580115.sapp.pdf]

PNAS
www.pnas.org

Supplementary Information for

Structure and architecture of immature and mature murine leukemia virus capsids

Kun Qu, Bärbel Glass, Michal Doležal, Florian K. M. Schur, Brice Murciano, Alan Rein, Michaela Rumlová, Tomáš Ruml, Hans-Georg Kräusslich, John A. G. Briggs

John A. G. Briggs
Email: jbriggs@mrc-lmb.cam.ac.uk

This PDF file includes:

- Figs. S1 to S8
- Tables S1 to S2
- Captions for movies S1 to S4

Other supplementary materials for this manuscript include the following:

- Movies S1 to S4

www.pnas.org/cgi/doi/10.1073/pnas.1811580115

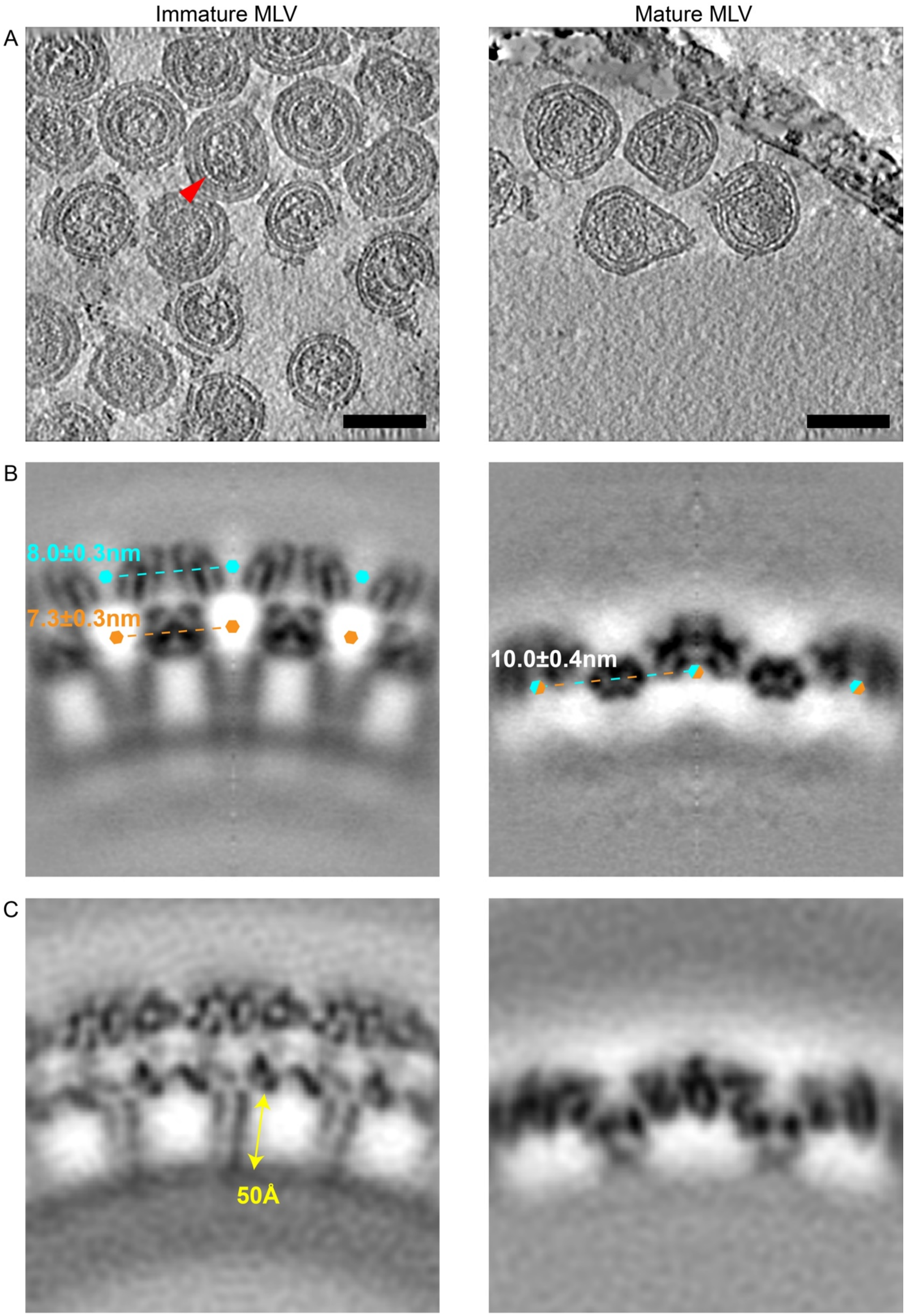
Immature MLV
Mature MLV
A
B
8.0±0.3nm
7.3±0.3nm
10.0±0.4nm
C
50Å

Fig. S1. Cryo-ET and subtomogram averaging reconstructions of immature and mature MLV CA lattices. (A) Computational slices through representative tomograms of immature and mature MLV. Density is black. Red arrowhead indicates the immature CAH bundles between CA and NC layers. Spiral and nested cores are distinctly recognizable in mature MLV particles. In many particles the membrane is disrupted. Scale bar: 100 nm. (B) Central orthoslices through unsharpened reconstructions of immature and mature MLV CA hexamers. Distinct curved layers corresponding to CA-NTD and CA-CTD are observed. Hexamer centers in the CA lattice are labelled with cyan (NTD) or orange (CTD) hexagons. The lattice spacing is marked by dashed lines. (C) Offset orthoslices through sharpened reconstructions for which the alignment was refocused on the downstream densities, to show the CAH densities on the six-fold axis (immature) and 3-fold axis (mature). The length of CAH densities in the immature virus that extend towards the RNP layer is marked.

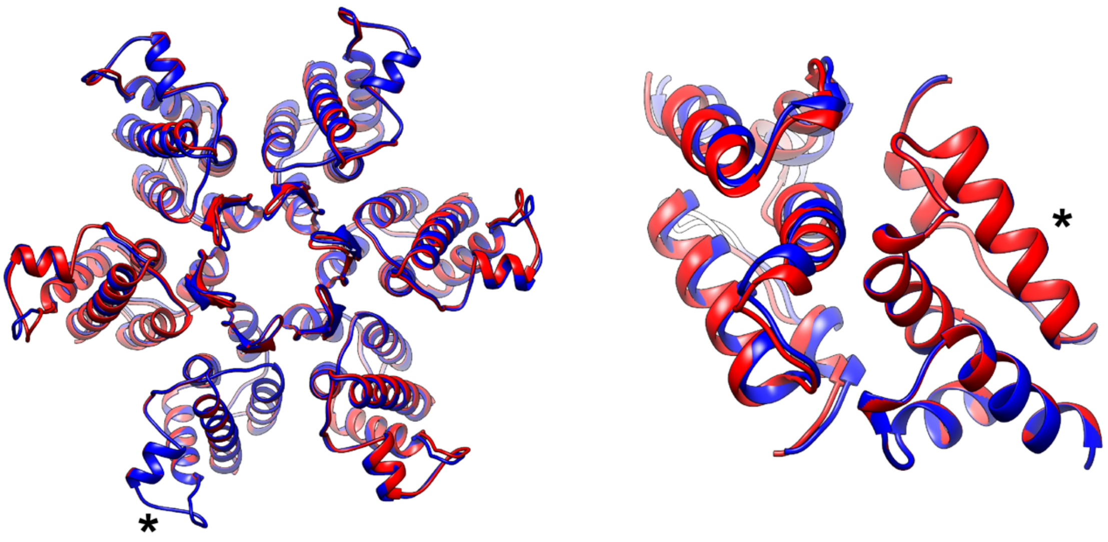

Mature MLV CA-NTD hexamer | Mature MLV CA-CTD dimer

Fig. S2. Structural comparison of mature MLV CA-NTD hexamer and CA-CTD dimer in the models derived from fitting into cryo-ET structures (blue, this study) and in the crystal structures (red, CA-NTD PDB ID 1U7K, CA-CTD this study) generated by UCSF Chimera MatchMaker. In both cases the alignment is performed using a single monomer marked with an asterisk. The crystallized CA-NTD hexamer and CA-CTD dimer correspond very well to the forms seen in the mature virion.

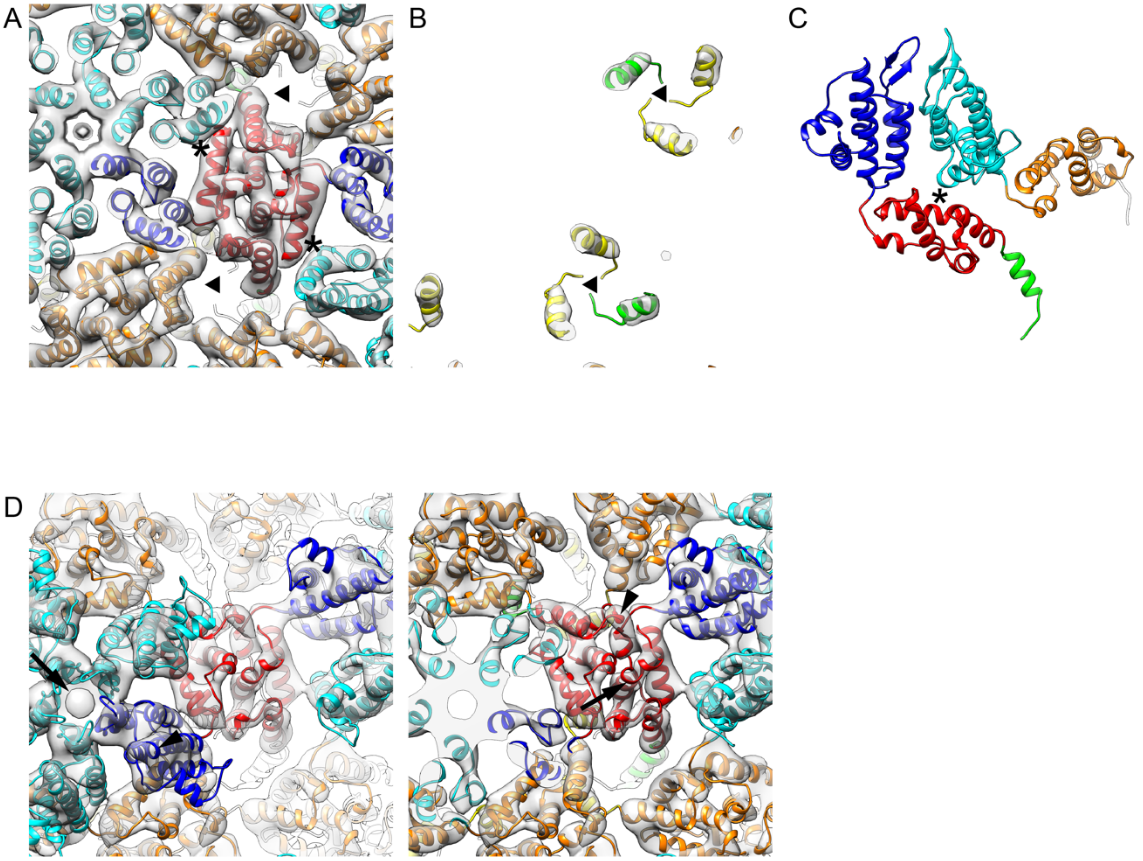

Fig. S3. Structural interactions in the mature MLV CA lattice. (A) As Fig. 3B for comparison. The three-fold symmetry axis is marked by triangles. The CAH tail in mature MLV is found at the three-fold axis. Asterisk indicates the CA-NTD - CA-CTD interface. (B) Close up image of the CAH layer in the mature MLV hexamer. The field of view is identical to A. (C) The interface between the CA-NTD and the neighboring CA-CTD is marked with an asterisk. (D) Exactly as described in Fig. 3, but for the mature pentamer.

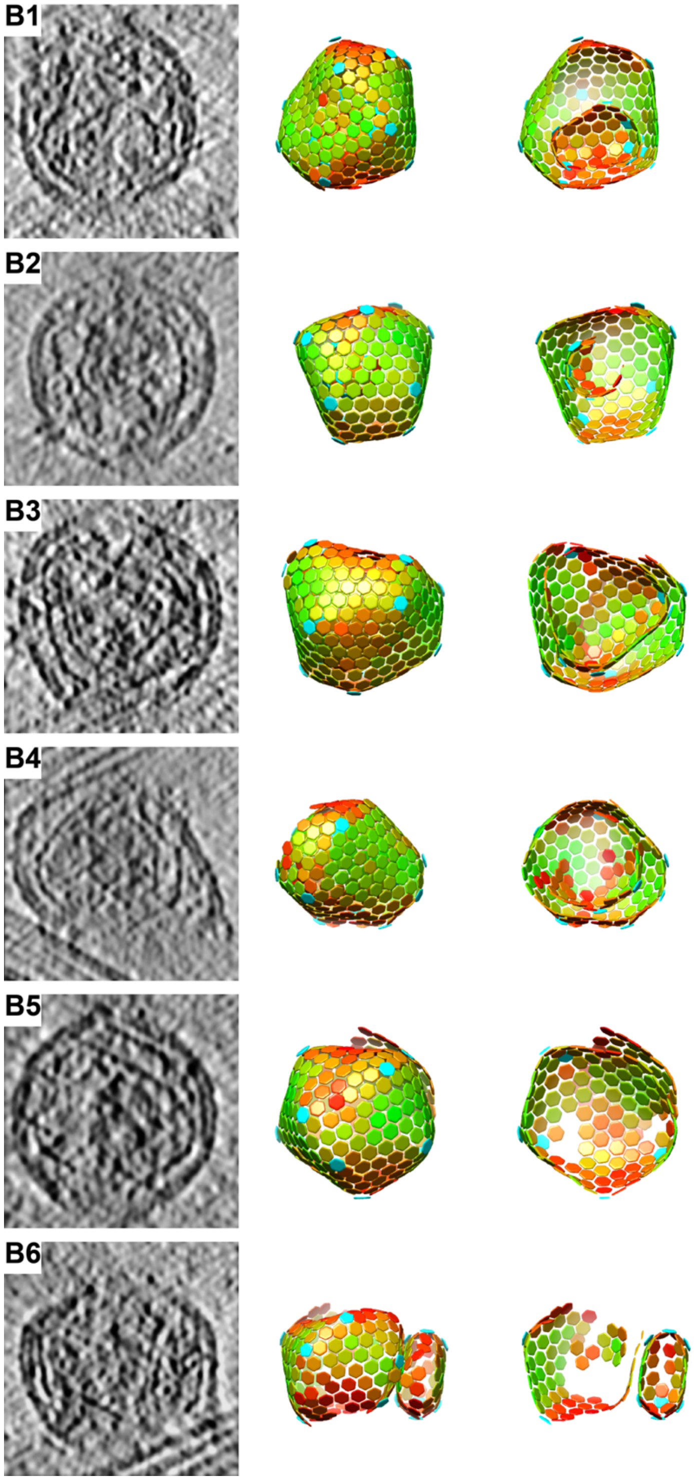

Fig. S4. Perpendicular (XZ) slices and lattice map views of the mature cores shown in Fig. 4B. The ability to identify the positions of mature hexamers is not strongly dependent on the orientation of the hexamer relative to the missing wedge.

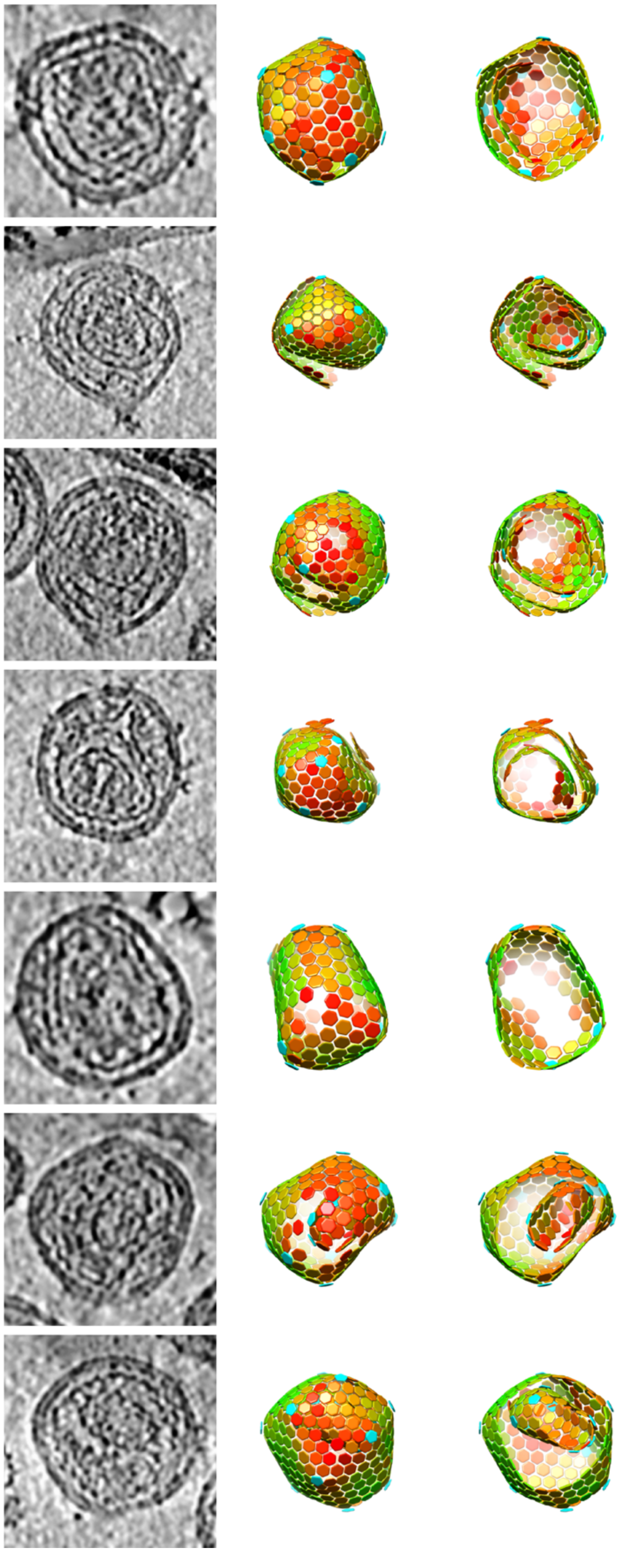

Fig. S5. Further examples of MLV mature core morphology. See Fig. 4 for explanation.

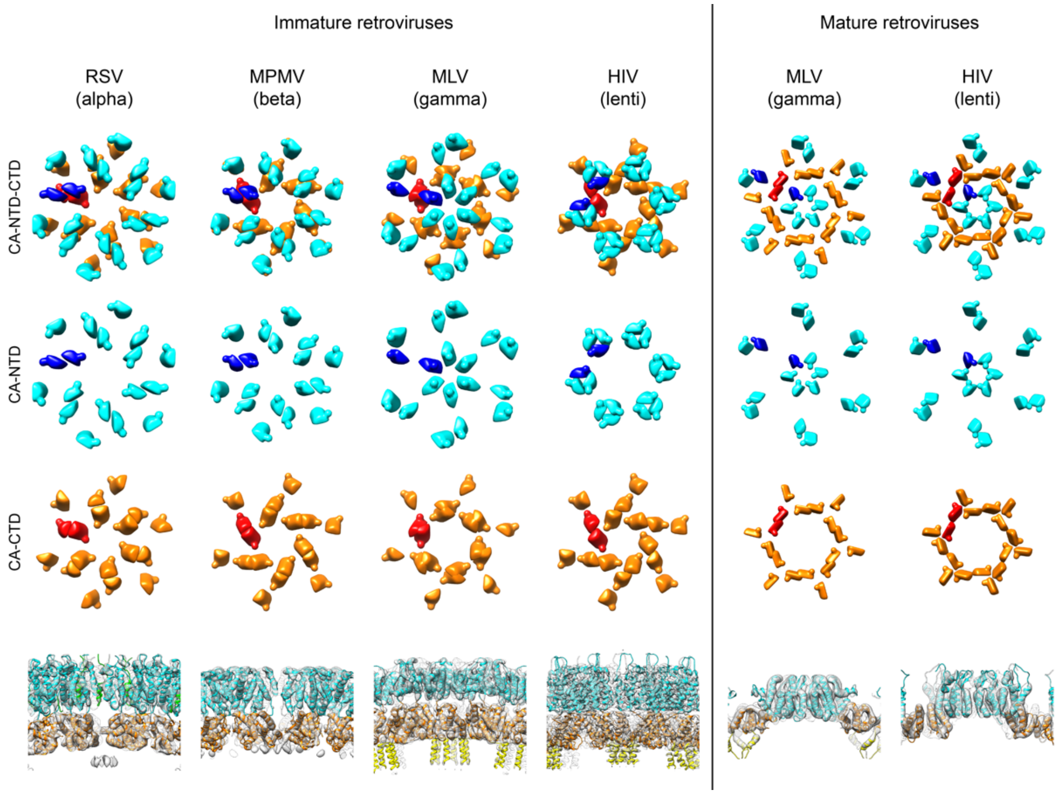

Fig. S6. Comparison of CA domain arrangements in immature and mature retroviruses. Schematic diagram in the top three panels represents the arrangements of the CA-NTD (cyan/blue) and CA-CTD (orange/red) domains. The bottom panel shows the side view of structures fitted with PDB models. CAH in MLV, and the CA-SP1 helix in HIV are colored yellow. The p10 peptide of RSV that forms part of the CA-NTD lattice is colored green.

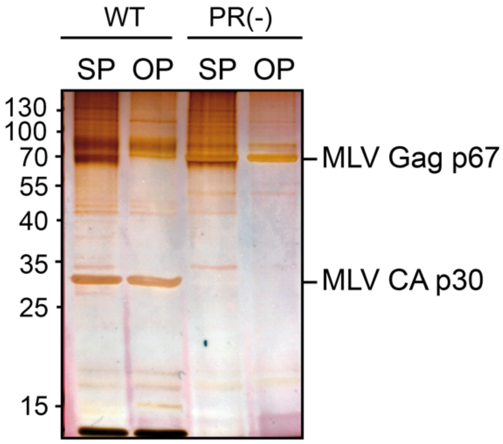

Fig. S7. MLV Gag and CA detected by SDS-PAGE. Particle preparations of wild-type and PR(-) MLV were separated by SDS-PAGE (12.5%) and visualized by silver staining according to standard procedures. SP indicates the preparation after concentration through a 20% (w/w) sucrose cushion, OP indicates the preparation after iodixanol gradient purification.

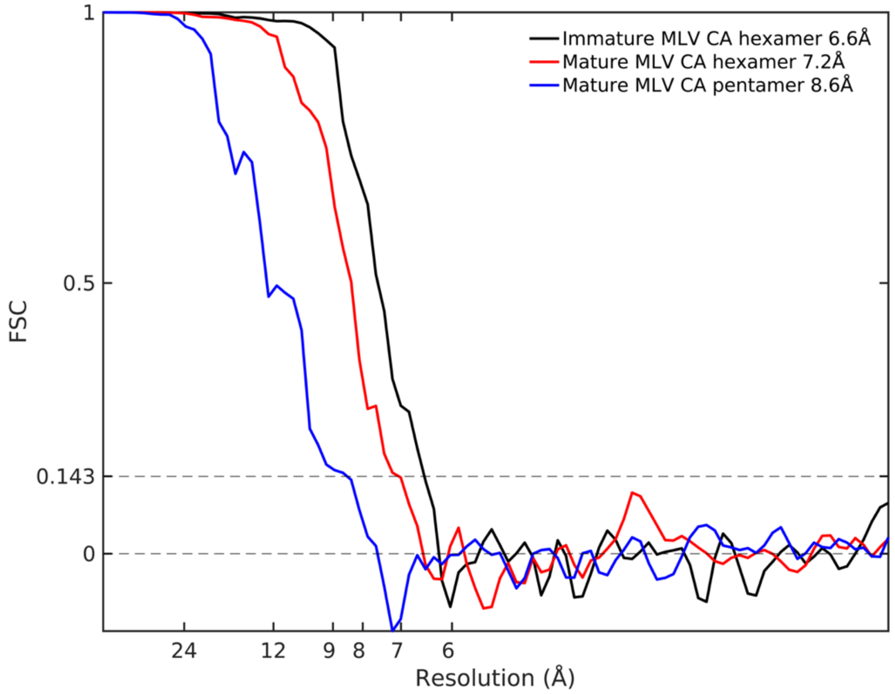

Fig. S8. Fourier shell correlation curves. FSC curves between the two independent half maps were calculated using cylindrical masks including one central hexamer or pentamer of CA. Resolution values were reported at the 0.143 threshold. Immature MLV hexamer (black), mature MLV hexamer (red), mature MLV pentamer (blue).

Table S1. X-ray data collection and refinement statistics for the MLV CA-CTDΔCAH crystal.

**Sample**	**MLV CTDΔCAH (132-218)**
**Data collection**	
Wavelength (Å)	1.5418
Space group	P 31 2 1
Unit Cell (Å)	71.198, 71.198, 77.068
Resolution (Å)	48.15-1.89 (1.99-1.89) [a]
R_{merge} (%)	0.100 (0.960) [a]
$CC_{1/2}$	0.999 (0.657) [a]
$\langle I/\sigma \rangle$	18.9 (2.0) [a]
Completeness (%)	100.0 (100.0) [a]
Redundancy	10.2 (7.5) [a]
**Refinement**	
Resolution (Å)	35.60-1.89
No. of reflections	34,992
R_{work}/R_{free}	0.1883/0.2297
No. of non-hydrogen atoms	
Protein	1,394
Co^{2+}	1
Water	171
B factors (Å^2)	
Protein	36.2
Co^{2+}	11.3
Waters	42.4
RMSD bond (Å)	0.01
RMSD angle (˚)	1.07
PDB ID	6GZA

Table S2. Cryo-EM data acquisition and image processing for MLV and M-PMV.

**Sample**	**Immature MLV**	**Mature MLV**		**Immature M-PMV**
**Data acquisition**				
Microscope	FEI Titan Krios	FEI Titan Krios		FEI Titan Krios
Voltage (keV)	300	300		300
Energy-filter (eV)	20	20		20
Detector	Gatan Quantum K2 Summit	Gatan Quantum K2 Summit		Gatan Quantum K2 Summit
Pixel size (Å)	1.35	1.35		1.35
Defocus range (microns)	-2.0 to -4.5	-2.0 to -7.5		-2.0 to -4.5
Defocus step (microns)	0.25	0.25		0.25
Acquisition scheme	-60/60°, 3°	-60/60° or -66/66°, 3°		-60/60°, 3°
Total Dose (electrons/Å^2)	~100	~50 to ~100 [a]		~70 to ~100 [a]
Dose rate (electrons/Å^2/sec)	~4.0	~2.2 to ~4.6 [a]		~1.8 to ~2.8 [a]
Frame number	6	5 or 6		5 or 6
Tomogram number	20	65		34
Tomogram EMDB ID	4422	4419		4421
**Image processing**				
Viral particles	79	134	134	71
Subtomogram (set A/B)	11,582/11,559	11,708/11,689	650/649	8,557/8,552
Symmetry	C6	C6	C5	C6
Resolution (0.143 FSC)	6.6 Å	7.2 Å	8.6 Å	7.2 Å
B factor	-677.5	-665.2	-748.7	-502.4
EMDB/PDB ID	0291/6HWW	0292/6HWX	0293/6HWY	0290/6HWI

[a] Dose fluctuation was caused by the ring collapse of FEG during data collection.

Movie S1. A tour of the CA region, showing electron density and fitted model, illustrating the features shown in Fig. 2 and Fig. 3. The movie shows the immature virus structure first, then morphs (as in Movie S4) into the mature virus structure.

Movie S2. Representative mature MLV CA spiral core. One continuously wrapped core is observed in a single virus. Hexameric and pentameric CA references are translated and oriented according to the parameters determined from subtomogram averaging and then displayed in a new plug-in for UCSF Chimera. The references are isosurfaces generated from the PDB models solved or refined in this study. CAH region is not shown.

Movie S3. Representative mature MLV CA nested core. Two complete nested cores are observed in one virus. See Movie S2 for more explanation.

Movie S4. Structural comparison between the immature and mature MLV CA lattice. The alignment is generated in UCSF Chimera with the morph command. Color scheme and PDB models used in this movie are identical to Fig. 2.
